# The second interim analysis of Italian participants enrolled in the real-world, Pan-European, prospective, observational, Phase 4 PEARL study of fremanezumab effectiveness

**DOI:** 10.1007/s10072-025-08504-0

**Published:** 2025-10-17

**Authors:** Fabrizio Vernieri, Anna Ambrosini, Marco Bartolini, Sabina Cevoli, Gianluca Coppola, Florindo D’Onofrio, Roberto De Simone, Antonio Granato, Simona Guerzoni, Maria Pia Prudenzano, Innocenzo Rainero, Renata Rao, Cristina Tassorelli, Pinar Kokturk, Mario Cepparulo

**Affiliations:** 1 Headache and Neurosonology Unit, Fondazione Policlinico Campus Bio-Medico and Università Campus Bio-Medico di Roma, Rome, Italy; 2 Headache Unit, IRCCS Neuromed, Pozzilli, Italy; 3 Neurological Clinic, Marche Polytechnic University, Via Conca 1, Ancona, Italy; 4 Istituto delle Scienze Neurologiche di Bologna, Programma Cefalee e Algie Facciali, Bologna, Italy; 5 Department of Medico-Surgical Sciences and Biotechnologies, Sapienza University of Rome Polo Pontino, ICOT, Latina, Italy; 6 Neurology Unit, San Giuseppe Moscati Hospital, Avellino, Italy; 7 Headache Centre, Department of Neuroscience RSO, University of Naples Federico II, Naples, Italy; 8 Clinical Unit of Neurology, Department of Medicine, Surgery and Health Sciences, Headache Centre, University Hospital and Health Services of Trieste - ASUGI, University of Trieste, Strada di Fiume, Trieste, Italy; 9 Department of Specialist Medicines, Digital and Predictive Medicine, Pharmacology and Clinical Metabolic Toxicology-Headache Center and Drug Abuse, Laboratory of Clinical Pharmacology and Pharmacogenomics, AOU Policlinico di Modena, Modena, Italy; 10 Headache Center, Clinical Unit of Neurology “L. Amaducci”, AOU Policlinico di Bari, Department of Translational Biomedicine and Neurosciences (DiBraiN), Bari, Italy; 11 Headache Center, Department of Neuroscience, University of Torino, Torino, Italy; 12 Department of Neurological Sciences and of Vision, P.le Spedali Civili, Brescia, Italy; 13 Department of Brain and Behavioral Sciences, University of Pavia, Pavia, Italy; 14 IRCCS C. Mondino Foundation, Pavia, Italy; 15 Teva Netherlands B.V., Haarlem, Netherlands; 16 Teva Italia Srl, Milan, Italy

**Keywords:** Calcitonin gene-related peptide, Fremanezumab, Migraine, Monoclonal antibodies, Real-world data, Real-world evidence

## Abstract

**Introduction:**

Migraine burden encompasses social, economic, and functional burden; most notably in individuals with either chronic or high-frequency episodic migraine (CM, HFEM). This second interim sub-analysis of Italian participants with CM and HFEM enrolled in the PEARL study provides updated real-world evidence of fremanezumab use in Italian clinical practice for up to 12 months of treatment.

**Methods:**

In this second interim sub-analysis, data were collected from a sub-population of participants enrolled in PEARL who were treated in Italian headache centers and had completed 12 months of fremanezumab treatment (data cut-off: 15 June 2023). The primary endpoint was the proportion of participants achieving a ≥ 50% reduction in monthly migraine days (MMD) during the 6-month period after fremanezumab initiation. Additional endpoints were assessed up to Month 12 and included mean change from baseline in average MMD. Safety was assessed through adverse events (AEs) reported.

**Results:**

Overall, 343/354 participants enrolled in Italian headache centers (HFEM, 36.2%; CM, 63.8%) were included in the effectiveness analysis. Of those with data available, 61.2% of participants achieved a ≥ 50% reduction in average MMD during the 6-month period after fremanezumab initiation. Mean change from baseline (± standard deviation [SD]) in average MMD was − 8.2 ± 7.4 at Month 1 and − 8.9 ± 6.9 at Month 12. No new safety signals were observed.

**Conclusion:**

Fremanezumab was effective and well tolerated in Italian clinical practice, with results in line with those previously reported for fremanezumab in a real-world setting.

**Supplementary Information:**

The online version contains supplementary material available at 10.1007/s10072-025-08504-0.

## Introduction

Migraine is a neurovascular disorder that affects 14% of people worldwide [1]. Its widespread impact leads to substantial social, economic, psychological, and functional burden [2–5]. Furthermore, several studies have identified bidirectional relationships between migraine and psychiatric, cardiovascular, and gastrointestinal disorders, increasing the overall burden of migraine [6–10].

Migraine is categorized into episodic migraine (EM; <15 monthly headache days [MHD]) and chronic migraine (CM; ≥15 MHD for > 3 months and ≥ 8 monthly migraine days [MMD]) by the International Classification of Headache Disorders (ICHD) diagnostic criteria (third revision) [11]. CM is associated with more headache-related disability, higher healthcare resource utilization, and lower health-related quality of life compared with EM [12]. However, many patients fluctuate above and below the 15 MHD threshold. Indeed, a large, longitudinal survey found that 73.4% of respondents with CM at baseline had at least one 3-month period where they did not meet CM diagnostic boundary of ≥ 15 MHD, and 7.6% of respondents with EM at baseline had at least one 3-month period where they met the headache frequency criteria for CM [13], suggesting that the classification of migraine does not fully capture the burden of illness or treatment needs of patients. Indeed, studies suggest that patients with a subset of EM defined as high-frequency episodic migraine (HFEM, 8–14 MHD) experience similar disease burden and disability as patients with CM [14, 15].

Calcitonin gene-related peptide (CGRP) pathway monoclonal antibodies (mAbs) are the first preventive treatments designed to target the underlying pathophysiology of migraine [16]. Fremanezumab, a mAb that potently and selectively binds to alpha-CGRP and beta-CGRP [16], has demonstrated efficacy in patient populations that are conventionally difficult to manage and who did not benefit from or tolerate multiple previous preventive medications [17–19]. In 2020, the Italian Medicines Agency (AIFA) approved the reimbursement of CGRP pathway mAbs, including fremanezumab, for the preventive treatment of migraine in patients with HFEM or CM [20]. To fulfill AIFA reimbursement criteria, patients must have ≥ 8 MMD, a Migraine Disability Assessment (MIDAS) questionnaire score of ≥ 11 points before treatment, and experience of ≥ 3 other preventive treatment failures, excluding other CGRP pathway treatments, due to lack of efficacy or tolerability. Furthermore, AIFA requires a ≥ 50% reduction from baseline in MIDAS score at Months 3 and 6 to authorize renewal of the prescription. Reimbursement is available for up to 12 months; preventive treatment can then be restarted after a minimum 1-month mandatory drug holiday if patients who meet the AIFA criteria experience worsening of migraine.

The Pan-European Real Life (PEARL; EUPAS35111) study is a multicenter, prospective, observational, non-interventional, Phase 4 study designed to evaluate the effectiveness, safety, and tolerability of fremanezumab in participants with CM or EM from 11 European countries in a real-world clinical setting. This study is the largest real-world data generation study of fremanezumab to date, with a varied cohort and a follow-up period of 24 months. In the first interim analysis of Italian participants enrolled in the PEARL study, fremanezumab treatment resulted in clinically relevant reductions in MMD, Headache Impact Test-6 (HIT-6) and MIDAS scores, and acute migraine medication use over 6 months, with no new safety signals identified [21]. In addition, the analysis supported the concept that reductions in MMD fail to fully capture the benefits of preventive treatment as, at Month 6, 61.2% of participants achieved a ≥ 50% reduction of MMD, while 81% achieved a ≥ 50% reduction in MIDAS score. This second interim analysis of the PEARL study, solely focused on Italian participants with HFEM and CM, aimed to present longer-term effectiveness and safety data over 12 months of fremanezumab treatment.

## Methods

The complete PEARL study protocol and the methodology used for the first interim analysis in Italian participants have been previously published [21, 22]. A summary of the methodology used for the second interim analysis in Italian participants is reported here.

### Study oversight

The protocol was approved by the Independent Ethics Committee or Institutional Review Board of all participating countries (Czech Republic, Denmark, Finland, Greece, Italy, Norway, Portugal, Spain, Sweden, Switzerland, and the United Kingdom). Local regulations, including relevant data protection laws, were followed. No further study procedures beyond participants’ routine clinical practice were performed for the duration of the study. Individual informed consent was obtained, and participants agreed that their clinical data could be anonymously recorded. Participants were aware of their right to withdraw their consent at any point during the study period [22].

### Participants and procedures

The first participant was screened and enrolled in the PEARL study in August 2020, and the last participant completed the 24-month observation period in March 2024. Enrollment in Italy started in February 2021. This second interim analysis was conducted after all 354 enrolled participants had completed 12 months of treatment (data cut-off: 15 June 2023).

All participants in the PEARL study were required to complete a daily headache diary as part of their routine disease management and were recommended to attend quarterly physician visits (every 3 months ± 15 days; nine visits overall) as part of routine clinical practice and at the discretion of their physician (**Supplementary Fig. 1**). Headache diary data were collected regardless of a missed clinic visit. Participants who discontinued fremanezumab treatment remained in the study, were documented for the entire observational period in local clinical practice and encouraged to continue to complete their headache diaries as per guidelines for routine disease management. Once discontinued, fremanezumab treatment could be resumed at the physician’s discretion. Participants were discontinued from the study if they were treated with a newly prescribed preventive migraine treatment after discontinuation of fremanezumab, were non-compliant to study procedures, or if they were lost to follow-up.

### Assessment of outcomes

The primary endpoint was the proportion of participants reaching a ≥ 50% reduction from baseline (the 28-day period prior to initiating fremanezumab treatment) in average MMD during the 6-month period after fremanezumab initiation. Additional endpoints in this interim analysis were assessed up to Month 12 and included the proportion of participants reaching a ≥ 50% reduction from baseline in average MMD, mean change from baseline in MMD, and mean changes from baseline in MIDAS score, HIT-6 score, acute medication use, and pain intensity (as reported by the Numerical Rating Scale [NRS]) score.

The MIDAS questionnaire is used to determine how severely migraine affects a participant’s life, with a scoring system assigned as: 0–5: little or no disability; 6–10: mild disability; 11–20: moderate disability; and > 20: severe disability [23]. The HIT-6 questionnaire quantifies the impact of migraine on a participant’s ability to function daily, with the scoring system as follows: 50–55: some impact; 56–59: substantial impact; and ≥ 60: severe impact on a participant’s life [24]. Clinically meaningful improvements in MIDAS and HIT-6 scores are defined as ≥ 4.5-point and 8-point reductions from baseline, respectively [25, 26].

The safety of fremanezumab, as reported through adverse events (AEs), was captured up to Month 12.

### Statistical methods

The full analysis set (FAS) included all enrolled participants from Italian centers with ≥ 10 days of data available between treatment initiation and the last documented follow-up visit. Effectiveness data from the FAS were analyzed using participant-reported outcomes measured from disability tools and participant diaries. The safety analysis set (SAS) included all participants who received fremanezumab treatment.

All variables are summarized descriptively, with continuous variables analyzed with descriptive statistics for their actual values and changes from baseline to each visit. For categorical variables, frequency and proportion are reported.

## Results

### Study population

**Fig. 1:**
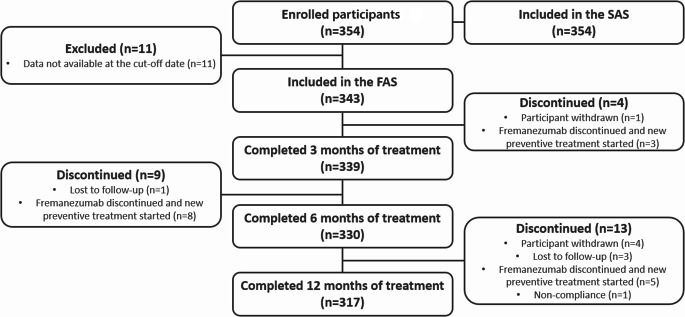
Participant disposition. *FAS* full analysis set, *SAS* safety analysis set

At data cut-off for the second interim analysis (15 June 2023), 31% (*n* = 354/1140) of participants enrolled in the PEARL study were from Italian centers. All participants were included in the SAS (Fig. 1), while 11 participants were excluded from the FAS due to data not being entered into the Electronic Data Capture system by the cut-off date. Of the 343 participants in the FAS, 26 (7.6%) discontinued the study within 12 months of treatment. Of these, 16 (61.5%) switched from fremanezumab to an alternative preventive treatment, five (19.2%) withdrew from the study, four (15.4%) were lost to follow-up, and one (3.8%) discontinued due to non-compliance with study procedures. Among the 26 participants who discontinued the study, 25 (7.3% of the FAS) permanently discontinued fremanezumab treatment. The most common reasons for permanently discontinuing treatment were lack of effectiveness (*n* = 16) and lack of tolerability (*n* = 6).

Most participants in the FAS were > 35–≤65 years of age (76.4%, *n* = 262), female (82.2%, *n* = 282), and had CM (63.8%, *n* = 219) (Table 1). Overall, 91% (*n* = 312) received monthly fremanezumab. As per Italian reimbursement criteria for CGRP pathway mAbs, all participants had preventive treatment failures in the last 5 years, with the most common being tricyclic antidepressants (90.7%, *n* = 311), anticonvulsants (83.7%, *n* = 287), and beta-blockers (71.1%, *n* = 244). Of the 90.4% (*n* = 310) of participants with data available, mean time from migraine onset to initiation of fremanezumab treatment was 29.6 ± 13.3 years. Most participants (98.8%, *n* = 339) had a MIDAS score of ≥ 11 points at baseline, indicating moderate or severe disability. The most frequently reported comorbidities were psychiatric disorders (24.2%, *n* = 83), metabolism and nutrition disorders (16.9%, *n* = 58), and vascular disorders (14.0%, *n* = 48). Gastrointestinal disorders were reported in 10.8% (*n* = 37) of participants. Concomitant medications for migraine prevention were utilized in a minority of participants, most commonly tricyclics (6.4%, *n* = 22), beta-blockers (5.8%, *n* = 20), and anticonvulsants (5.5%, *n* = 19).

**Table 1:** Participant baseline demographics and clinical characteristics

	Overall population (*N* = 343)
Sex female, n (%)	282 (82.2)
Age (years), mean ± SD	48.3 ± 12.2
Age (years), n (%)	
18–34	45 (13.1)
35–44	74 (21.6)
45–54	118 (34.4)
55–64	70 (20.4)
65–≥75	36 (10.5)
Weight (kg), mean ± SD	64.0 ± 12.0
BMI (kg/m^2^), mean ± SD	23.1 ± 3.6
Chronic migraine, n (%)	219 (63.8)
Migraine duration (years), mean ± SD	29.6 ± 13.3
MMD, mean ± SD	15.6 ± 6.6
Monthly average days with any acute headache medication use, mean ± SD	13.0 ± 6.5
MIDAS score, mean ± SD	93.9 ± 62.9
HIT-6 score, mean ± SD	67.4 ± 4.9
Fremanezumab administration, n (%)	
Monthly	312 (91.0)
Quarterly	12 (3.5)
Both doses	19 (5.5)
Prior preventive treatments in the last 5 years by drug class, n (%)	
Angiotensin II receptor antagonists	11 (3.2)
Anticonvulsant medications (excluding valproic acid)	287 (83.7)
Beta-blockers	244 (71.1)
Calcium channel blockers	204 (59.5)
Erenumab	13 (3.8)
Galcanezumab	2 (0.6)
OnabotulinumtoxinA	104 (30.3)
Tricyclic antidepressants	311 (90.7)
Valproic acid	64 (18.7)
Concomitant medication use, n (%)	
Angiotensin II receptor antagonists	1 (0.3)
Antiseizure	19 (5.5)
Beta-blockers	20 (5.8)
Calcium channel blockers	1 (0.3)
Tricyclic antidepressants	22 (6.4)
Valproic acid	3 (0.9)
Other preventive migraine medications	9 (2.6)

*BMI* body mass index, *HIT-6* Headache Impact Test-6, *MIDAS* Migraine Disability Assessment, *MMD*, monthly migraine days, *SD* standard deviation

### Primary endpoint

**Fig. 2:**
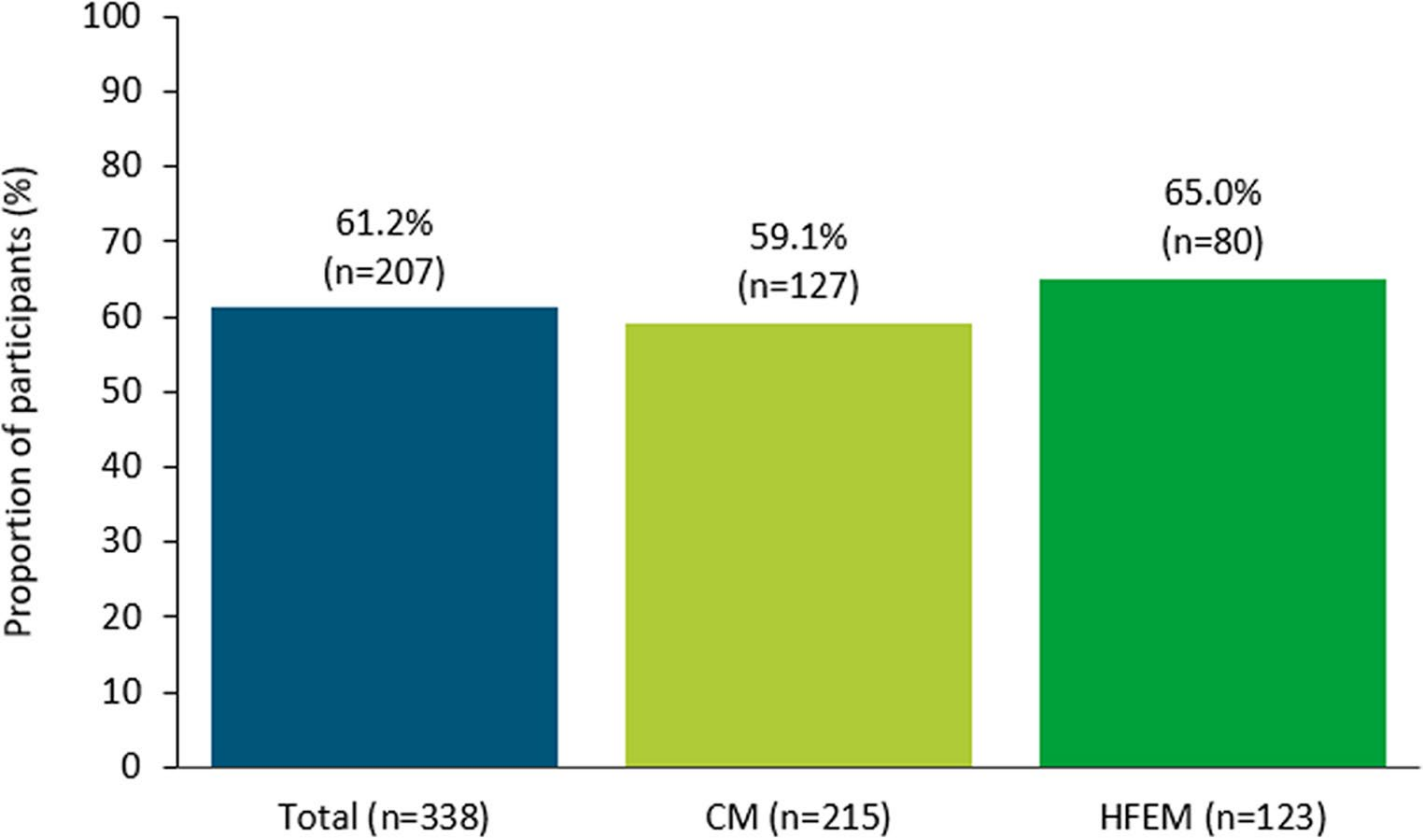
Proportion of participants reaching ≥ 50% reduction in average MMD over 6 months of fremanezumab treatment by migraine type. *CM* chronic migraine, *HFEM* high-frequency episodic migraine, *MMD* monthly migraine days

Data for the primary endpoint were available for 338 participants (98.5%). Of those, 207 (61.2%) achieved a ≥ 50% reduction in MMD during the 6-month period after fremanezumab initiation, with the proportion of responders slightly higher in participants with HFEM (65.0%, *n* = 80) versus CM (59.1%, *n* = 127; Fig. 2).

### Additional endpoints

#### MMD

**Fig. 3:**
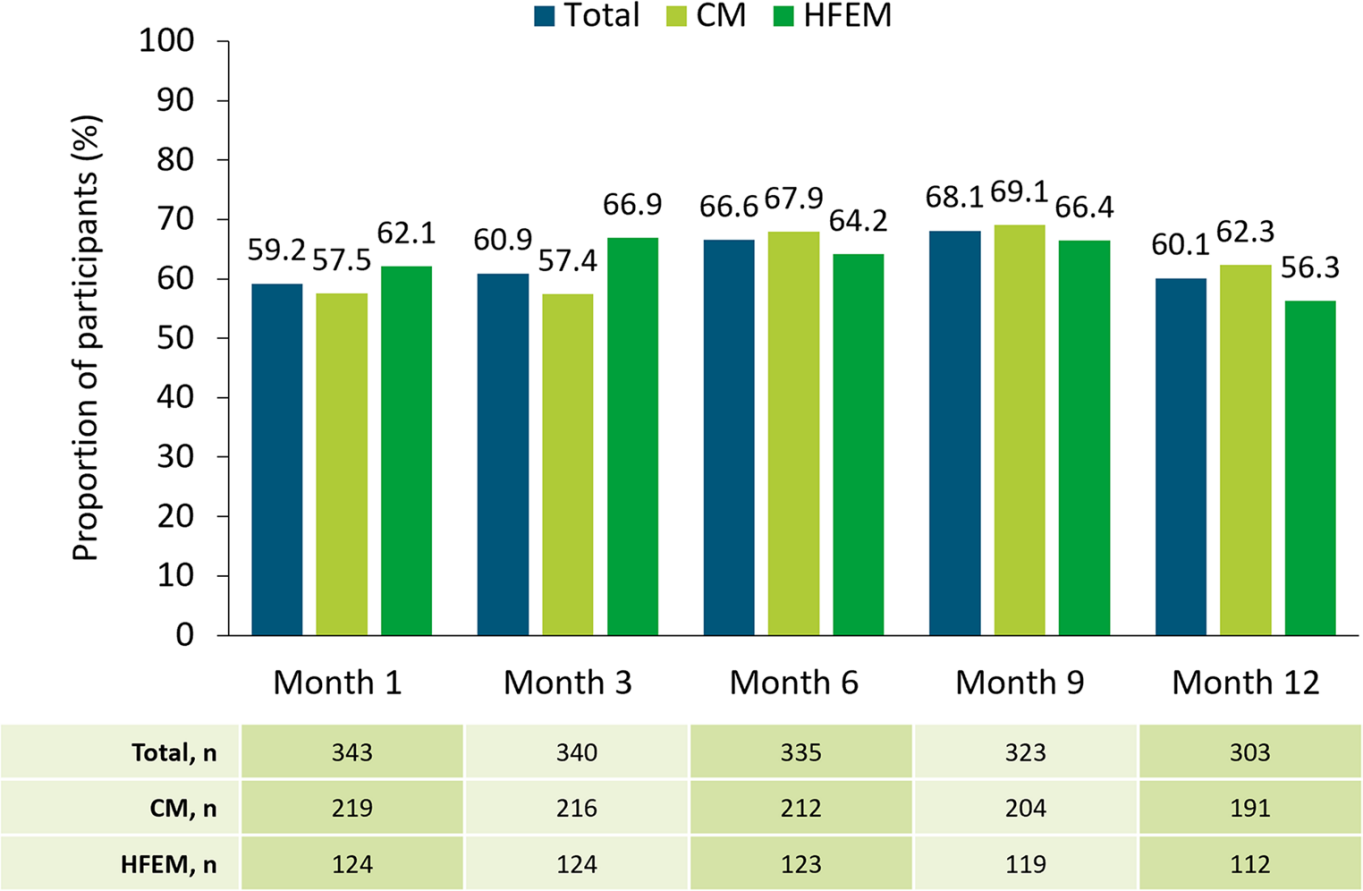
Proportion of participants reaching ≥ 50% decrease in average MMD by migraine type at Month 1, 3, 6, 9, and 12. The table reports participant numbers for each month. The drop in n numbers at each time-point are due to not all data for this endpoint being available at data cut-off, missing data being excluded, and delays in data being entered into the electronic data capture system. *CM* chronic migraine, *HFEM* high-frequency episodic migraine, *MMD* monthly migraine days

Among participants with data available, the proportion who achieved a ≥ 50% reduction in MMD was 59.2% (*n* = 203/343) at Month 1, 60.9% (*n* = 207/340) at Month 3, 66.6% (*n* = 223/335) at Month 6, and 60.1% (*n* = 182/303) at Month 12 (Fig. 3).

**Fig. 4:**
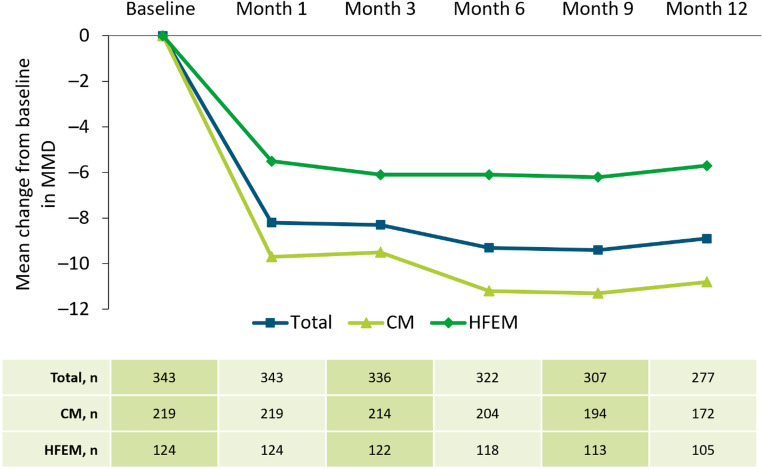
Change from baseline in MMD at Month 1, 3, 6, 9, and 12 by migraine type. The table reports participant numbers for each month. The drop in n numbers at each time-point are due to not all data for this endpoint being available at data cut-off, missing data and data from participants prematurely discontinuing the study being excluded, and delays in data being entered into the electronic data capture system. *CM* chronic migraine, *HFEM* high-frequency episodic migraine, *MMD* monthly migraine days

The mean change from baseline (± SD) in average MMD at Months 1 and 12 was − 8.2 ± 7.4 and − 8.9 ± 6.9 for the overall population, − 9.7 ± 8.2 and − 10.8 ± 7.5 for participants with CM, and –5.5 ± 4.6 and − 5.7 ± 4.2 for participants with HFEM, respectively (Fig. 4).

#### Migraine-related disability and pain intensity scores

Overall, 75.3% (*n* = 195/259) of participants achieved a reduction of ≥ 50% in MIDAS score from baseline to Month 12, with the largest proportion of participants (80.8%, *n* = 256/317) achieving this endpoint at Month 6. At Month 12, 80.0% (*n* = 72/90) of participants with HFEM and 72.8% (*n* = 123/169) of participants with CM achieved a ≥ 50% reduction in MIDAS score (**Supplementary Fig. 2**). The mean change from baseline (± SD) in MIDAS score at Month 12 was − 66.7 ± 61.1 for the overall population, − 75.1 ± 68.6 for CM, and − 51.5 ± 40.5 for HFEM (**Supplementary Fig. 3**).

The mean change from baseline (± SD) in HIT-6 score at Month 12 was − 11.0 ± 10.0 for the overall population, − 11.1 ± 9.7 for CM, and − 10.8 ± 10.7 for HFEM (**Supplementary Fig. 4**).

The mean change from baseline (± SD) in NRS score for remaining migraine attacks decreased at each time-point from − 1.1 ± 1.5 at Month 1 to − 1.6 ± 1.8 at Month 12. The mean change from baseline (± SD) in NRS score at Months 1 and 12, respectively, was − 1.0 ± 1.5 and − 1.4 ± 1.6 for CM, and − 1.2 ± 1.6 and − 1.9 ± 1.9 for HFEM.

#### Acute migraine medication use

The mean change from baseline (± SD) in monthly average number of days with acute migraine medication use was sustained from Month 1 to Month 12 (Month 1: −7.9 ± 6.4 days; Month 12: −8.1 ± 6.2 days; **Supplementary Fig. 5**). In general, participants with CM experienced a larger decrease from baseline in monthly average number of days with acute migraine medication use versus participants with HFEM (Month 1: − 9.0 ± 7.2 vs. − 5.9 ± 4.0; Month 12: –9.6 ± 6.9 vs. − 5.6 ± 4.0; **Supplementary Fig. 5**).

Among participants who used triptans at baseline, 70.6% (*n* = 173/245) and 60.4% (*n* = 137/227) achieved a ≥ 50% reduction in mean monthly days of triptan use at Months 1 and 12, respectively.

### Safety

Of the 354 participants included in the SAS, 90 (25.4%) reported ≥ 1 AE (HFEM: 25.6%; CM: 25.3%) and 51 (14.4%) reported a treatment-related AE. The most common treatment-related AEs by system organ class were general disorders and administration site conditions (9.9%, *n* = 35), and gastrointestinal disorders (4.0%, *n* = 14). Of those reporting gastrointestinal disorders, 10 (2.8%) participants reported constipation and four (1.1%) reported nausea during 12 months of treatment. There were no reports of hypertension during this period (Table 2). Only six participants permanently discontinued treatment due to intolerance.

**Table 2:** Participants with treatment-related AEs at Month 12

	Overall participants, SAS (*N* = 354)
Participants with treatment-related AE, n (%)	51 (14.4)
General disorders and administration site conditions, n (%) Drug ineffective Injection site erythema Injection site pruritus Injection site edema Injection site swelling	35 (9.9) 12 (3.4) 12 (3.4) 8 (2.3) 4 (1.1) 4 (1.1)
Gastrointestinal disorders, n (%) Constipation Nausea	14 (4.0) 10 (2.8) 4 (1.1)
Nervous system disorders, n (%) Dizziness	8 (2.3) 4 (1.1)
Skin and subcutaneous tissue disorders, n (%)	5 (1.4)
Infections and infestations, n (%)	3 (0.9)
Psychiatric disorders, n (%)	1 (0.3)
Musculoskeletal and connective tissue disorders, n (%)	1 (0.3)
Respiratory, thoracic, and mediastinal disorders, n (%)	1 (0.3)
Reproductive system and breast disorders, n (%)	1 (0.3)
Immune system disorders, n (%)	1 (0.3)
Ear and labyrinth disorders, n (%)	1 (0.3)

Specific disorders are reported in ≥ 1% of participants

*AE* adverse event, *SAS* safety analysis set

## Discussion

The second Italian interim analysis of the PEARL study took place when all enrolled Italian participants (*n* = 354) had completed 12 months of treatment with fremanezumab. Over half of participants with CM (59.1%) and HFEM (65.0%) reached ≥ 50% reduction in average MMD during the first 6 months of treatment. Fremanezumab effectiveness was further demonstrated throughout the study in clinically relevant reductions from baseline in MMD, HIT-6, MIDAS, and NRS scores, alongside reductions in acute migraine medication use. Fremanezumab was well tolerated, no new safety signals were observed, and the proportion of participants reporting constipation AEs was lower than those observed in real-world evidence (RWE) studies of CGRP-receptor targeting therapies [27–29]. The results of this analysis are similar to those observed in previous PEARL interim analyses and are supported by RWE studies in Italy, Greece, and the United States, which all corroborate the effectiveness and safety of fremanezumab in real-world settings [21, 28, 30–34].

RWE has demonstrated that a substantial proportion of patients treated with CGRP pathway mAbs are late- (≤ 6 months) and ultra-late (≤ 12 months) responders [35, 36]. In this analysis, a higher proportion of participants with HFEM achieved a ≥ 50% reduction in MMD during the first 6 months of fremanezumab treatment versus participants with CM. These data suggest that patients with CM may require a longer duration of treatment with fremanezumab to achieve a similar response as patients with HFEM, which may be attributed to pathophysiological differences in these conditions [37]. This latter hypothesis is supported by the increase in the proportion of participants with CM reaching ≥ 50% reduction in MMD at Month 9 compared with Month 6, although responder selection bias cannot be excluded with certainty. These findings not only support the effectiveness of fremanezumab in clinical practice, but also suggest that in this difficult-to-treat population, a longer treatment period may be required to achieve greater responses. Moreover, more timely preventive treatment with fremanezumab in patients with HFEM may have the potential to prevent progression to CM.

While reductions in MMD are often used as the primary efficacy measure of preventive therapies for migraine [11], other measures, including clinician and patient-reported outcomes, are important for examining the full extent of treatment benefit [38]. Indeed, the AIFA requires a ≥ 50% reduction from baseline in MIDAS score at Months 3 and 6 to allow patients to continue CGRP pathway mAb treatment [20]. In this analysis, treatment with fremanezumab was associated with a high proportion of participants reaching ≥ 50% reductions in MIDAS scores at Month 3 and Month 6, in line with the AIFA reimbursement criteria. Participants also achieved sustained reductions in MIDAS and HIT-6 scores at Month 12, suggesting that the residual migraine days were less severe and had a reduced impact on participants’ disability and quality of life compared with migraine days prior to enrollment. In addition, reductions in acute medication use were observed over 12 months, with slight variations between HFEM and CM, indicating that treatment with fremanezumab may reduce the risk of medication overuse. The combination of multiple endpoints in this study may provide a more accurate representation on the benefits of fremanezumab in relieving the burden of migraine.

CGRP pathway mAbs have been shown to be well tolerated in randomized controlled trials (RCTs) [16–18, 39]. As the PEARL study contains a heterogenous participant population that is representative of patients seen in clinical practice, it offers an opportunity to observe the tolerability of fremanezumab outside the regulated environment of an RCT. A recent analysis of records from the Food and Drug Administration Adverse Event Reporting System (FAERS) database identified general disorders and administration site conditions, and injury, poisoning, and procedural complications, as the most reported AEs for all CGRP pathway mAbs [40]. The overall occurrence of treatment-related AEs in this interim analysis of the PEARL study was low, and, in alignment with the FAERS study, the most common AEs were general disorders and administration site conditions (9.9%), followed by gastrointestinal disorders (4.0%), suggesting that fremanezumab is well tolerated in real-world clinical settings [17, 40].

CGRP pathway-targeting preventive migraine treatments, notably those targeting the CGRP receptor, have been shown to be associated with constipation and hypertension AEs in real-world settings, highlighting the relevance of collecting long-term data [27, 40, 41]. Constipation was the most reported AE in a 2-year prospective study of 160 patients with CM treated with erenumab, occurring in 34 patients (21.2%) following 6 months of treatment, with severe constipation leading to treatment discontinuation in 11 patients by Month 6, and in a further four patients by Month 12 [27]. Constipation was also reported more frequently than anticipated for atogepant in the FAERS database, with a reporting odds ratio of 12.86 (the threshold for this measure was ≥ 3) [40]. In a 12-month prospective study of erenumab and fremanezumab, patients receiving erenumab experienced significant systolic and diastolic blood pressure increases at all measured time-points versus baseline (*p* < 0.001). In comparison, patients receiving fremanezumab experienced systolic but not diastolic blood pressure increases at Month 3 (*p* = 0.006) and Month 6 (*p* = 0.004) only. Four patients (3.7%) with normal blood pressure at baseline required antihypertensive treatment after receiving erenumab [42]. An analysis of the FAERS database identified 61 cases of elevated blood pressure associated with erenumab treatment between 2018 and 2020, which led to the inclusion of hypertension in the Warnings and Precautions section of the erenumab prescribing information [41]. The incidence of constipation in this interim analysis of the PEARL study was low (2.8%), and there were no reports of hypertension, supporting the potential difference between CGRP receptor-targeting and CGRP ligand-targeting treatments.

A key strength of this study is the real-world setting. Although RCTs are a key component of evidence-based medicine, they do not always reflect real-world participant populations, limiting their generalizability and external validity [43]. RWE can assess effectiveness in routine clinical practice, with regulatory bodies including the FDA recognizing its value for clinical and regulatory decision-making [44]. This interim analysis of the PEARL study explores the effectiveness and safety of fremanezumab across 30 sites in Italy, suggesting that the data gathered has a broad applicability in clinical practice.

It is important to note that the outcomes reported in this analysis relied on participant self-diaries, which represents a source of bias as outcomes may be misrepresented due to human error. At data cut-off, not all data for certain endpoints were available, and missing data were excluded. The number of participants who prematurely discontinued the study (in addition to delays in data being entered into the electronic data capture system) contributed to the drop in numbers of participants available for analysis, which particularly affected results at Month 12.

Additionally, as this study was initiated during the coronavirus disease pandemic (COVID-19), there was limited access to participants, and some gaps in data have occurred as a result. Finally, although it would be useful to explore any differences between monthly and quarterly dosing schedules of fremanezumab, only 3.5% of participants enrolled in this analysis used quarterly fremanezumab dosing.

## Conclusion

Data from the second interim analysis of the Italian cohort from the PEARL study build on the results from the first interim analysis, demonstrating that fremanezumab is effective in reducing the overall impact and burden of migraine, including migraine-related disability and utilization of acute medications, whilst maintaining a tolerable safety profile through 12 months of treatment. Notably, results from this analysis indicate that response to fremanezumab treatment is maintained over 12 months. Future PEARL analyses will further explore the effectiveness, tolerability, and safety of fremanezumab over longer periods of time.

## Supplementary Information

Below is the link to the electronic supplementary material.

Supplementary Material 1 (DOCX 354 KB)

## Data Availability

Qualified researchers may request access to patient level data and related study documents including the study protocol and the statistical analysis plan. Requests will be assessed for scientific merit, product approval status, and conflicts of interest. If the request is approved, patient level data will be de-identified and study documents will be redacted to protect the privacy of trial participants and to protect commercially confidential information. Please email USMedInfo@tevapharm.com to make your request.
