# Supplementary material for: The second interim analysis of Italian participants enrolled in the real-world, Pan-European, prospective, observational, Phase 4 PEARL study of fremanezumab effectiveness
Source: Neurol Sci. 2025 Oct 17;46(12):6895–905. doi: 10.1007/s10072-025-08504-0 (PMC12678496; doi:10.1007/s10072-025-08504-0)
Supplement: Supplementary file 1 — Supplementary Material 1 (DOCX 354 KB) [file 10072_2025_8504_MOESM1_ESM.docx]

**The second interim analysis of Italian participants enrolled in the real-world, Pan-European, prospective, observational, Phase 4 PEARL study of fremanezumab effectiveness**

**Neurological Sciences**

Fabrizio Vernieri^1^, Anna Ambrosini^2^, Marco Bartolini^3^, Sabina Cevoli^4^,
Gianluca Coppola^5^, Florindo D'Onofrio^6^, Roberto De Simone^7^, Antonio Granato^8^,
Simona Guerzoni^9^, Maria Pia Prudenzano^10^, Innocenzo Rainero^11^, Renata Rao^12^,
Cristina Tassorelli^13,14^, Pinar Kokturk^15^, Mario Cepparulo^16^

^1^Headache and Neurosonology Unit, Fondazione Policlinico Campus Bio-Medico and Università Campus Bio-Medico di Roma, Rome, Italy

^2^Headache Unit, IRCCS Neuromed, Pozzilli, Italy

^3^Neurological Clinic, Marche Polytechnic University, Via Conca 1, Ancona, Italy

^4^Istituto delle Scienze Neurologiche di Bologna, Programma Cefalee e Algie Facciali, Bologna, Italy

^5^Department of Medico-Surgical Sciences and Biotechnologies, Sapienza University of Rome Polo Pontino, ICOT, Latina, Italy

^6^Neurology Unit, San Giuseppe Moscati Hospital, Avellino, Italy

^7^Headache Centre, Department of Neuroscience RSO, University of Naples Federico II, Naples, Italy

^8^Clinical Unit of Neurology, Department of Medicine, Surgery and Health Sciences, Headache Centre, University Hospital and Health Services of Trieste - ASUGI, University of Trieste, Strada di Fiume, Trieste, Italy

^9^Department of Specialist Medicines, Digital and Predictive Medicine, Pharmacology and Clinical Metabolic Toxicology-Headache Center and Drug Abuse, Laboratory of Clinical Pharmacology and Pharmacogenomics, AOU Policlinico di Modena, Modena, Italy

^10^Headache Center, Clinical Unit of Neurology “L. Amaducci”, AOU Policlinico di Bari, Department of Translational Biomedicine and Neurosciences (DiBraiN), Bari, Italy

^11^Headache Center, Department of Neuroscience, University of Torino, Torino, Italy

^12^Department of Neurological Sciences and of Vision, P.le Spedali Civili, Brescia, Italy

^13^Department of Brain and Behavioral Sciences, University of Pavia, Pavia, Italy

^14^IRCCS C. Mondino Foundation, Pavia, Italy

^15^Teva Netherlands B.V., Amsterdam, Netherlands

^16^Teva Italia Srl, Milan, Italy

**Corresponding author:** Fabrizio Vernieri

Headache and Neurosonology Unit, Fondazione Policlinico Campus Bio-Medico and Università Campus Bio-Medico di Roma, Rome, Italy

**e-mail:** [F.Vernieri@policlinicocampus.it](mailto:F.Vernieri@policlinicocampus.it)

**ORCID-ID:** 0000-0002-9594-9336

**On behalf of the PEARL Italian study group:** Anna Ambrosini, Monica Bandettini, Piero Barbanti, Marco Bartolini, Chiara Benedetto, Filippo Brighina, Sabina Cevoli, Alberto Chiarugi, Gianluca Coppola, Roberto De Simone, Florindo D’Onofrio, Fabio Frediani, Pierangelo Geppetti, Sara Gori, Antonio Granato, Simona Guerzoni, Rosario Iannacchero, Stefano Messina, Francesco Perini, Maria Pia Prudenzano, Innocenzo Rainero, Renata Rao, Ester Reggio, Antonio Russo, Simona Sacco, Paola Sarchielli, Giuliano Sette, Susanna Usai, Cristina Tassorelli, Mariarosaria Valente, and Fabrizio Vernieri.

**Supplementary Figures**

**Supplementary Fig. 1** Scheme of study design and follow-up showing 28-days baseline and 24-months fremanezumab treatment

**
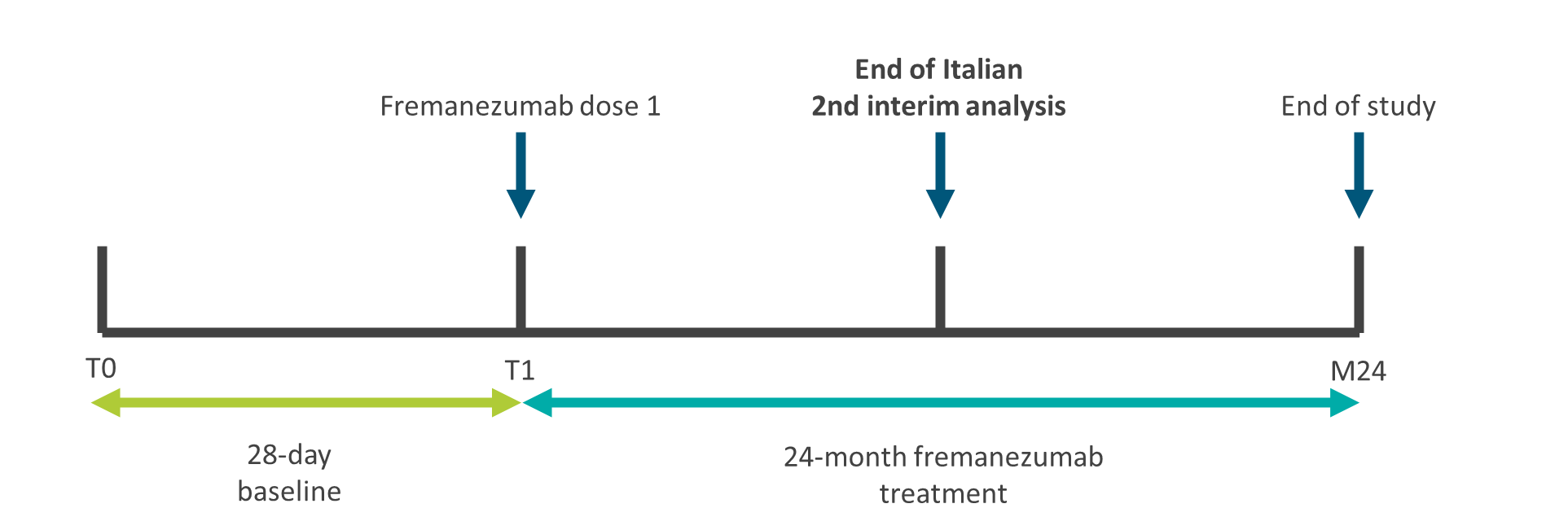
**

*M* months, *T* treatment

**Supplementary Fig. 2** Proportion of participants achieving a ≥50% reduction in MIDAS score compared to baseline by migraine type at Month 3, 6, 9, and 12

**
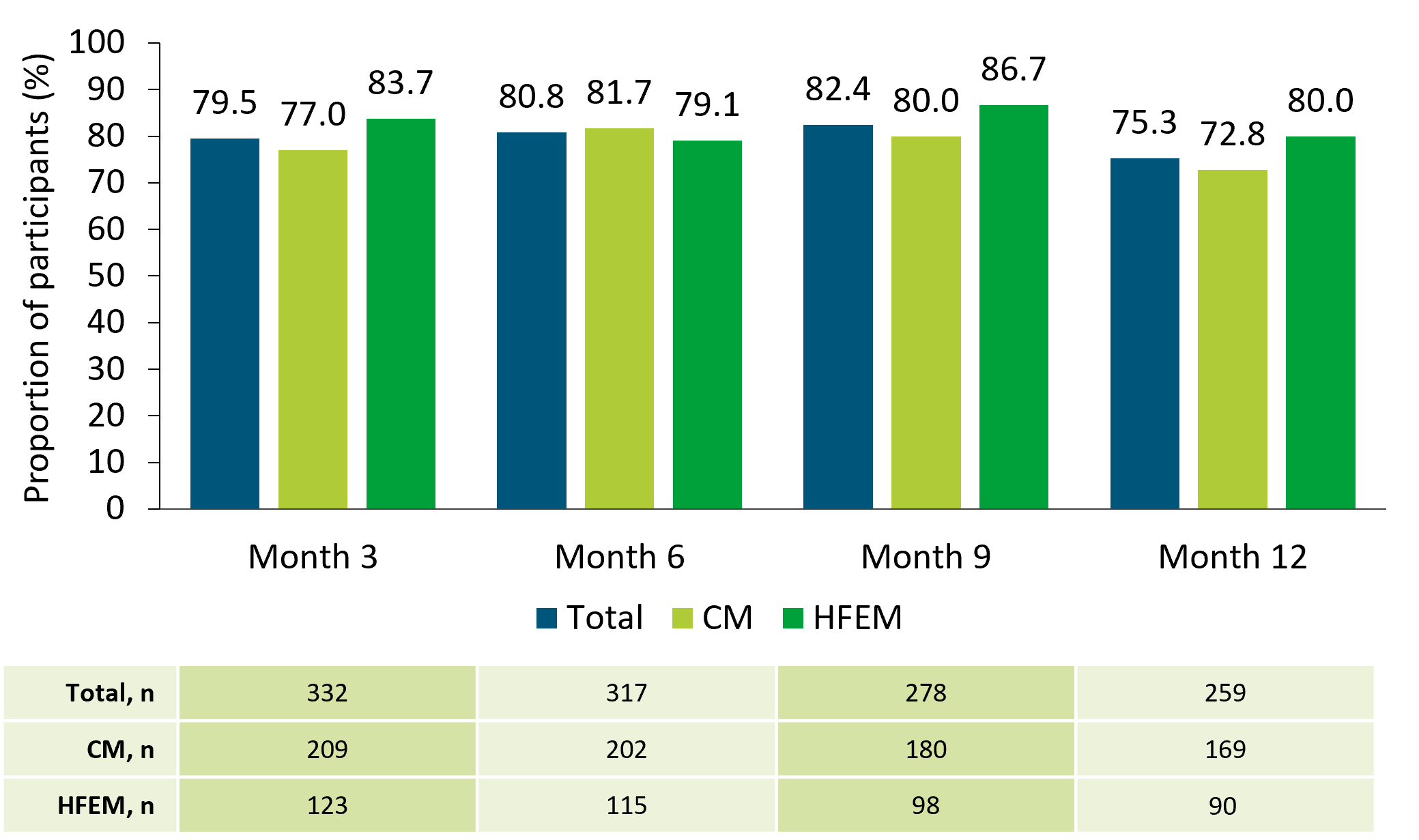
**

The table reports participant numbers for each month. The drop in n numbers at each time point are due to not all data for this endpoint being available at data cut off, missing data being excluded, and delays in data being entered into the electronic data capture system.
*CM* chronic migraine, *HFEM* high-frequency episodic migraine, *MIDAS* Migraine Disability Assessment

**Supplementary Fig. 3** Change from baseline in MIDAS score at Month 3, 6, 9, and 12 by migraine type


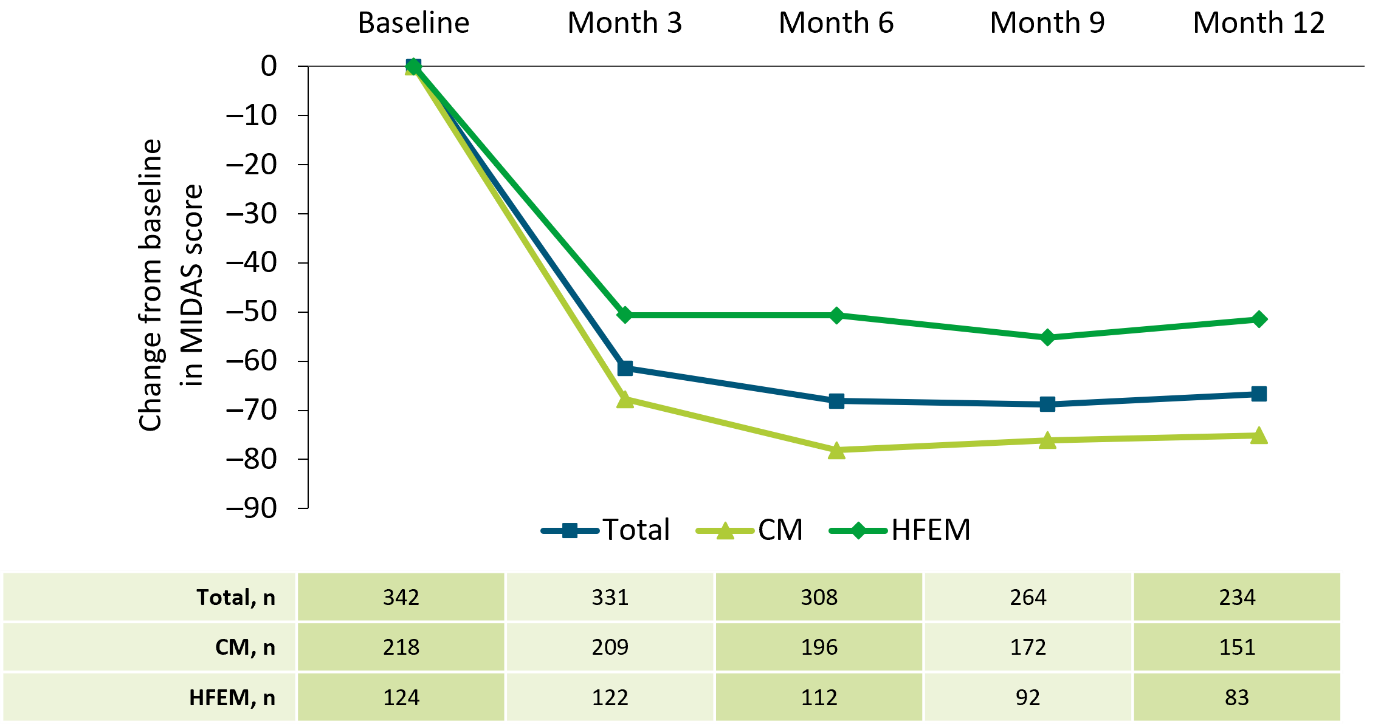


The table reports participant numbers for each month. The drop in n numbers at each time point are due to not all data for this endpoint being available at data cut off, missing data and data from participants prematurely discontinuing the study being excluded, and delays in data being entered into the electronic data capture system.
*CM* chronic migraine, *HFEM* high-frequency episodic migraine, *MIDAS* Migraine Disability Assessment

**Supplementary Fig. 4** Change in HIT-6 score from baseline by migraine type at Month 3, 6, 9, and 12


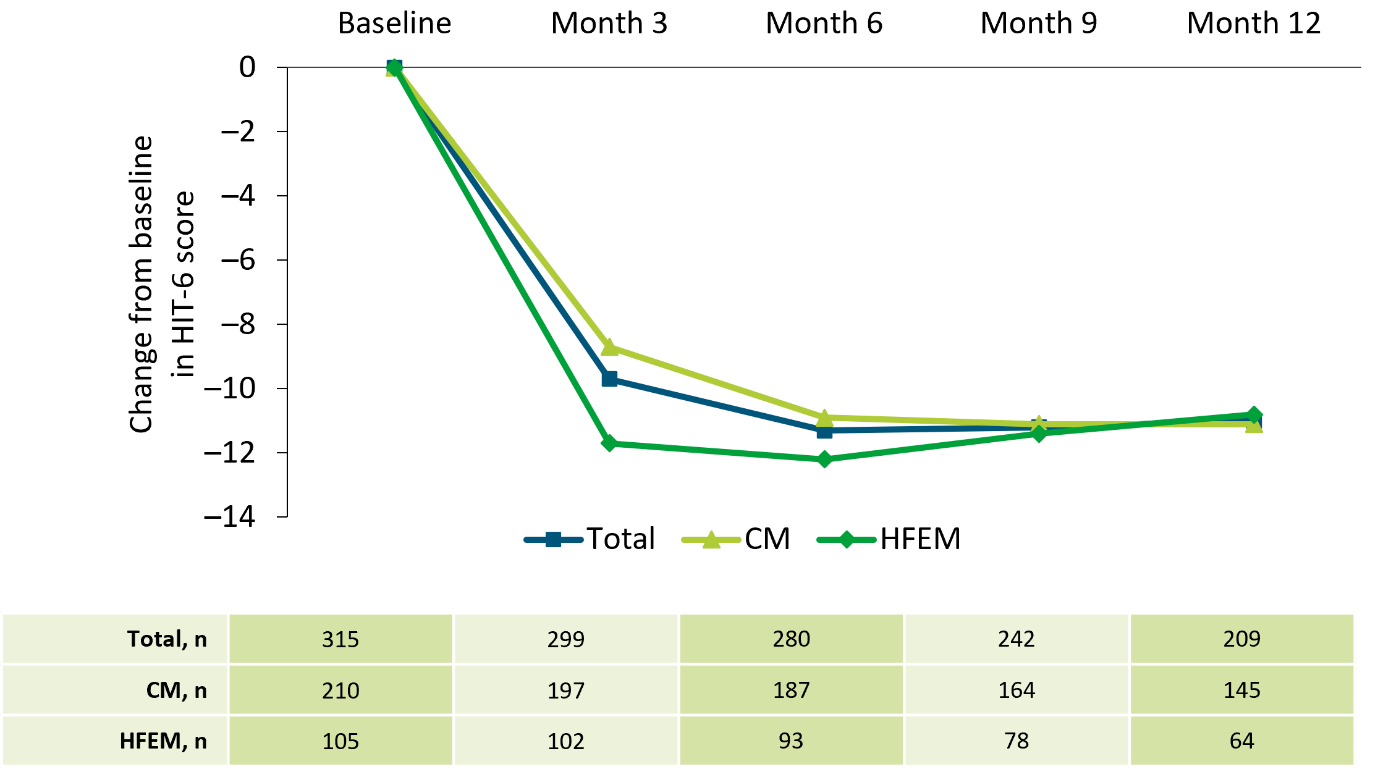


The table reports participant numbers for each month. The drop in n numbers at each time point are due to not all data for this endpoint being available at data cut off, missing data and data from participants prematurely discontinuing the study being excluded, and delays in data being entered into the electronic data capture system.
*CM* chronic migraine, *HFEM* high-frequency episodic migraine, *HIT-6* Headache Impact Test-6

**Supplementary Fig. 5** Change in average number of days from baseline with acute medication use (all analgesics) by migraine type at Month 1, 3, 6, 9, and 12

**
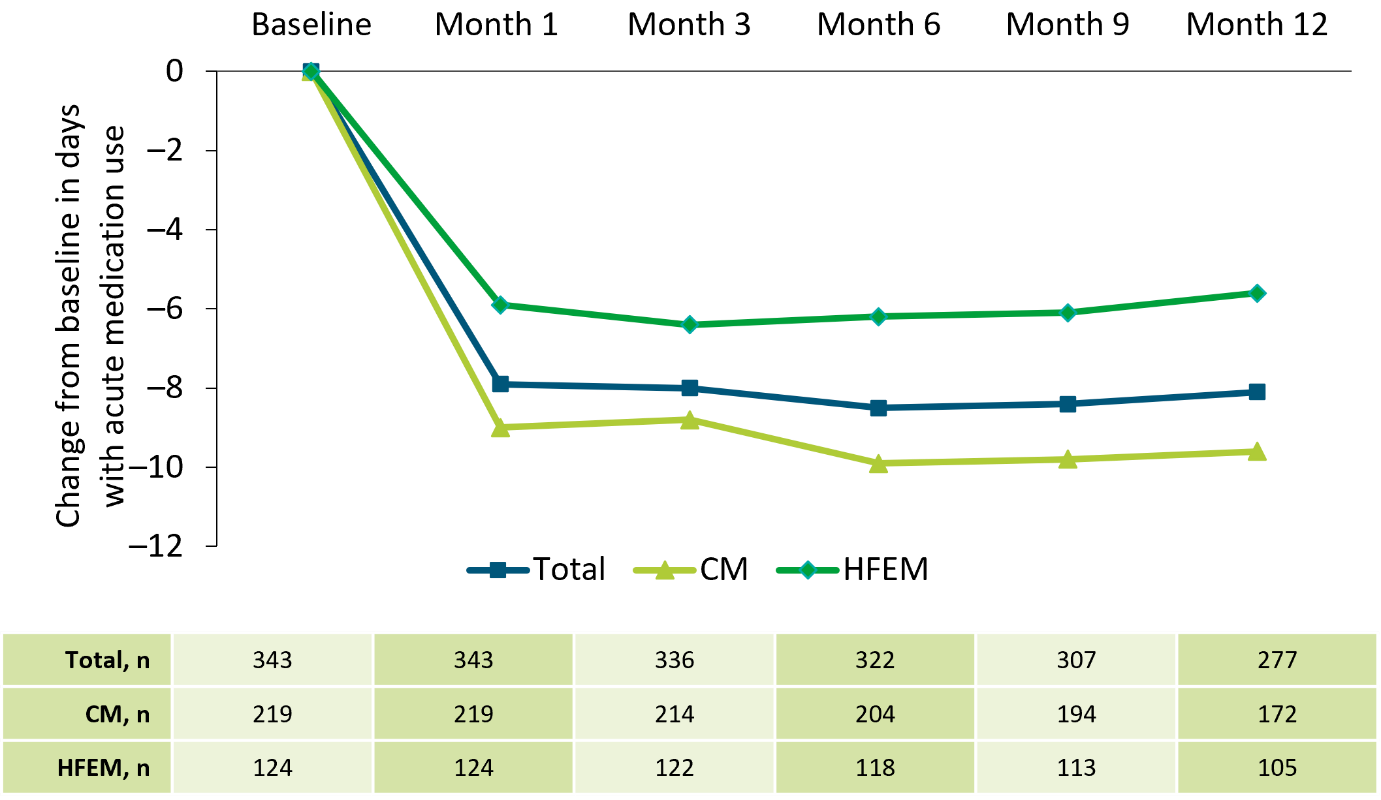
**

The table reports participant numbers for each month. The drop in n numbers at each time point are due to not all data for this endpoint being available at data cut off, missing data and data from participants prematurely discontinuing the study being excluded, and delays in data being entered into the electronic data capture system.
*CM* chronic migraine, *HFEM* high-frequency episodic migraine
